# Hypericum for Convulsions

**Published:** 1895-12

**Authors:** A. S. Ironside

**Affiliations:** Camden, New Jersey


### HYPERICUM FOR CONVULSIONS.

A. S. Ironside, M. D., Camden, New Jersey.

Mr. R—— called at the office at 12.15 A. M. July 10th, and stated that his nephew, four years old, was lying in convulsions. During the evening the boy, while playing with the other children, was running around a table and struck the right parietal region of his head upon the corner. He cried for some time, but was finally quieted and was apparently well the remainder of the evening.

At 11.15 P. M. the same night Mrs. R—— was awakened by a strange noise in her room, and upon going to the injured boy's bed found him in convulsions. The family waited until 12 P. M. thinking the struggling would stop, but this did not occur.

I sent Hypericum$^{cm}$, a powder to be dissolved in a few teaspoonfuls of water and a few drops to be given every ten minutes until convulsions stopped.

At 12.40 A. M. the first dose was given, the child being yet in violent spasms. Before the time for the second dose the eyes became straight and the lids closed. By one o'clock the little fellow was comfortable. In the morning he appeared to be in usual health and remains so.

